# Prevalence of Irritable Bowel Syndrome and Frequency of Symptoms in the General Population of Pakistan

**DOI:** 10.7759/cureus.12541

**Published:** 2021-01-06

**Authors:** Parkash Bachani, Love Kumar, Naresh Kumar, Maham Fatima, Sidra Naz, Muhammad Khizar Memon, Sidra Memon, Amber Rizwan

**Affiliations:** 1 Internal Medicine, Liaquat University of Medical and Health Sciences, Jamshoro, PAK; 2 Internal Medicine, Jinnah Sindh Medical University, Karachi, PAK; 3 Internal Medicine, University of Health Sciences, Lahore, PAK; 4 Internal Medicine, Liaquat University of Medical and Health Sciences, Hyderabad, PAK; 5 Family Medicine, Jinnah Post Graduate Medical Center, Karachi, PAK

**Keywords:** irritable bowel syndrome, prevalence, pakistan, symptoms

## Abstract

Introduction: Irritable bowel syndrome (IBS) is a chronic and debilitating functional gastrointestinal disorder. Risk factors include infective enteritis, female sex, antibiotic exposure, anxiety, and depression. The aim of this study is to find out the prevalence of IBS in healthy population and determine the characteristics of symptoms.

Material and Methods: A cross-section study was conducted in the internal medicine unit of a tertiary care hospital in multiple cities of Pakistan. Eight hundred (800) healthy peoples were selected for study from June 2019 to August 2019. Diagnosis of IBS was made by using Rome III criteria.

Results: The prevalence of irritable bowel syndrome (IBS) in general population in our study was 33.2%. IBS was more common in females compared to males (57.7% vs. 42.2%; p value = 0.009). IBS was more common in age group between 20 and 29 years (45.5%). Among patient diagnosed with IBS in this study, the most common was bloating (74.7%) followed by increased stool frequency (54.4%).

Conclusion: IBS is very prevalent in Pakistan, yet there is very little data and awareness related to it. Any change in stool frequency or consistency in young adults, especially women, shall be evaluated for IBS after ruling out other diseases. Early diagnosis and treatment of IBS will assist in improving the patient’s quality of life.

## Introduction

Irritable bowel syndrome (IBS) refers to a functional gastrointestinal disorder that has its chronic effects, including impact quality of life, on people across the world. Many theories have been postulated about the pathogenesis of this disorder; however, the exact pathophysiology is still uncertain [1]. Some studies suggest that a number of factors might be involved in the development of IBS, such as dysfunctional gastrointestinal motility, bacterial overgrowth, visceral hypersensitivity, altered gut flora, malabsorption, and inflammation [2]. Psychological aspects such as stress may also increase the severity of the symptoms associated with IBS [2]. According to a meta-analysis, among the several risk factors of IBS development, infective enteritis (IE) is the most important factor [3]. Other risk factors include female sex, antibiotic exposure, and presence of anxiety or depression [3].

IBS causes a variety of different symptoms in different patients, but the most common gastrointestinal symptoms include abdominal pain, bloating, diarrhea, and constipation. Extraintestinal symptoms are not uncommon, with fatigue being the most prevalent [2]. Patients diagnosed with IBS show a relatively poor quality of life and tend to utilize more health-care facilities as compared to people without the disorder [2].

The prevalence of IBS varies throughout the world. This variance partly depends upon the criteria that are used to diagnose this disorder. A meta-analysis pooled 53 studies from across the world, which had used the Rome III criteria and discovered that the pooled prevalence was 9.2%. However, when six (06) studies from 34 countries that used the Rome-IV diagnostic criteria were pooled, the prevalence was found to be 3.8% [4]. A study done in 2005 suggested that the prevalence of IBS in the USA was 14.1% [5]. The countrywide prevalence of IBS in Pakistan is not known, but a study conducted by college students in Karachi, Pakistan, showed that the prevalence of IBS in Karachi was 34% [6]. There is very limited data available on prevalence and characteristics of IBS in Pakistan. The goal of this study is to determine the prevalence of IBS on healthy population and identify the common symptoms associated with IBS.

## Materials and methods

This cross-section study was conducted in outpatient departments of internal medicine unit in tertiary care hospitals, of different cities in Pakistan, from June 2019 to August 2019. Eight hundred (800) healthy individuals were enrolled in study, through consecutive convenient non-probability sampling. These healthy individuals were attendants of patients coming to outpatient departments in hospitals. Approval was taken from ethical review board, before study.

After obtaining their consent, the patient’s age and gender were noted in self-structured questionnaire. Diagnosis of IBS was based on Rome III criteria [7]. Rome III criteria defines IBS as recurrent abdominal pain or discomfort for at least three (03) days per month during last three (03) months associated with two or more of the following symptoms: improvement with defecation, onset associated with a change in the frequency of stool, and onset associated with a change in the form (appearance) of stool.

The questionnaire was explained to participants, and the interviewer filled the answer. Participants that screened positive for IBS were asked details of their symptoms. Participants who screened negative were used as reference group, to compare characteristics, with IBS-positive cases.

Statistical analysis was done using SPSS v. 24.0 (IBM Corporation, Armonk, New York, United States). Continuous variables were analyzed via descriptive statistics and were presented as means and standard deviations (SDs) while categorical. Chi-square was applied to compare IBS-positive and IBS-negative groups. p value of less than 0.05 meant that the difference between the groups is significant, and the null hypothesis is void.

## Results

The prevalence of IBS in our study was 33.2%. IBS was more common in females compared to males (57.7% vs. 42.2%; p value, 0.009). IBS was more common in age group between 20 and 29 years (45.5%) (Table 1).

**Table 1:** Characteristics of IBS-Positive and IBS-Negative Participants IBS, Irritable bowel syndrome.

Characteristics	IBS Positive (n = 123)	IBS Negative (n = 277)	p value
Gender			
Male	52 (42.2%)	156 (56.3%)	0.009
Female	71 (57.7%)	121 (43.6%)	
Age (%)			
Younger than 20	21 (17.0%)	41 (14.8%)	<0.00001
20-29	56 (45.5%)	61 (22.02%)	
30-39	36 (29.2%)	51 (18.4%)	
40+	10 (8.1%)	124 (44.7%)	

Most common symptom was bloating (74.7%), followed by stool more than three per day (54.4%) (Table 2).

**Table 2:** Frequency of Symptoms in IBS-Positive Participants IBS, Irritable bowel syndrome.

Irritable Bowel Syndrome (IBS) Symptoms	Frequency	Percentages
Abdominal pain relieved by passing stool	51	41.4
Stool more than three per day	67	54.4
Stool less than three per week	38	30.8
Loose or watery stool	57	46.3
Mucus in stool	21	17.0
Bloating	92	74.7

## Discussion

The IBS is a common chronic disorder affecting 9%-23% of the population [8]. It is characterized by abdominal pain, bloating, cramps, diarrhea or constipation, or both. The diagnosis exclusively relies on the identification of the distinctive symptoms and exclusion of other organic disorders [9].

According to this study, IBS was more common in females. It was common in the age group of 20-29 years. Most common symptom reported in this study was bloating, followed by stool more than three per day.

IBS being the most prevalent functional disorder is affecting patients throughout the world. In this study, the prevalence of IBS in general population was 33.2%. High prevalence of IBS was reported in various other countries such as Croatia (28.2%), Iceland (30.9%), and Nigeria (31.6%) [10]. Female gender, young age, and gastrointestinal infection are the common risk factors in association with IBS [11]. Lovell et al. in her study concluded that IBS was more prevalent in female gender, similar to our findings [12]. Lovell et al. in another study found that IBS is more prevalent in people younger than 50 years [13].

Guidelines for the management of IBS advocate that a diagnosis can be done on clinical grounds, and there is no need for multiple investigations to rule out other organic diseases [14,15]. Treatment approach may include lifestyle and dietary modifications together with medication [16]. Dietary modification may include low-FODMAP (fermentable oligo-, di-, mono-saccharides, and polyols) diet, fiber supplementation, reducing gas-producing foods such as beans, onions, carrots, bananas, etc. Gluten-free diet or lactose-free diet may also benefit patients. Medication may include tricyclic antidepressants, antispasmodics, selective serotonin reuptake inhibitors (SSRIs), rifaximin, loperamide, cholestyramine, and laxatives [16]. Behavioral therapy may include cognitive behavioral therapy, hypnotherapy, and acupuncture [17].

Patients with IBS have poor quality of life and may add significant burden to health care, if not timely and properly diagnosed [1]. Hence it is important that general population, particularly young patients, should be regularly screened for IBS. They should be made aware of the symptoms and risk factors of IBS to assist clinicians in early diagnosis.

This study determines the prevalence of IBS in healthy general population in Pakistan. Previous studies were done on selected group of patients. There were limitations to this study as well. Since it is a cross-sectional study, risk factors for IBS were not identified, and association between the risk factors and incidence of IBS could not be established.

## Conclusions

In this study, prevalence of IBS in Pakistan is very high. Young females are commonly affected by IBS due to multiple reasons, especially psychosocial stressors. Young people should be screened for IBS. Any symptom of change in stool frequency or consistency should raise a suspicion of IBS after undergoing all the relevant investigations for ruling out organic diseases. This will aid their early diagnosis and will help to improve their quality of life.
